# Adverse Birth Outcomes as Indicators of Poor Fetal Growth Conditions in a French Newborn Population—A Stratified Analysis by Neighborhood Deprivation Level

**DOI:** 10.3390/ijerph16214069

**Published:** 2019-10-23

**Authors:** Wahida Kihal-Talantikite, Pauline Le Nouveau, Pierre Legendre, Denis Zmirou Navier, Arlette Danzon, Marion Carayol, Séverine Deguen

**Affiliations:** 1 LIVE UMR 7362 CNRS (Laboratoire Image Ville Environnement), University of Strasbourg, 6700 Strasbourg, France; 2 EHESP School of Public Health, 35043 CEDEX Rennes, France; pauline.le-nouveau@eleve.ensai.fr (P.L.N.); legendrepierre@LIVE.fr (P.L.); severine.deguen@ehesp.fr (S.D.); 3 INSERM U1085 IRSET (Research Institute in Environmental and Occupational Health), 35000 CEDEX Rennes, France; zmirou.denis@orange.fr; 4 Lorraine University Medical School, 54052 CEDEX Nancy, France; 5 City of Paris Maternal and infant health department (PMI), 75018 Paris, France; arlette.danzon@paris.fr (A.D.); marion.carayol@paris.fr (M.C.); 6 Department of Social Epidemiology, Sorbonne Universités, UPMC Univ Paris 06, INSERM, Institut Pierre Louis d’Epidémiologie et de Santé Publique (UMRS 1136), 75646 Paris, France

**Keywords:** adverse birth outcomes, fetal growth conditions, structural equation models (sem), neighborhood deprivation, social inequalities

## Abstract

*Background*: Adverse birth outcomes are related to unfavorable fetal growth conditions. A latent variable, named Favorable Fetal Growth Condition (FFGC), has been defined by Bollen et al., in 2013; he showed that this FFGC latent variable mediates the effects of maternal characteristics on several birth outcomes. *Objectives*: The objectives of the present study were to replicate Bollen’s approach in a population of newborns in Paris and to investigate the potential differential effect of the FFGC latent variable according to the neighborhood socioeconomic level. *Methods*: Newborn health data were available from the first birth certificate registered by the Maternal and Child Care department of the City of Paris. All newborns (2008–2011) were geocoded at the mother residential census block. Each census block was assigned a socioeconomic deprivation level. Several mothers’ characteristics were collected from the birth certificates: age, parity, education and occupational status and the occupational status of the father. Three birth outcomes were considered: birth weight (BW), birth length (BL) and gestational age (GA). *Results*: Using a series of structural equation models, we confirm that the undirected model (that includes the FFGC latent variable) provided a better fit for the data compared with the model where parental characteristics directly affected BW, BL, and/or GA. However, the strength, the direction and statistical significance of the associations between the exogenous variables and the FFGC were different according to the neighborhood deprivation level. *Conclusion:* Future research should be designed to assess the how robust the FFGC latent variable is across populations and should take into account neighborhood characteristics to identify the most vulnerable group and create better design prevention policies.

## 1. Introduction

Adverse birth outcomes (as low birth weight or preterm birth) remain a major public health concern due to the well documented association with neonatal mortality as well as both short- and long-term morbidity [1]. In 2015, about one million deaths among children under 5 years old were related to preterm birth complications [2]. Some studies investigated the relation between PTB, SGA and neonatal mortality [3,4] and suggested that small for gestational age (SGA) was associated with increased risks of stillbirth and neonatal mortality [5].

Several authors advanced that adverse birth outcomes could be related to unfavorable fetal growth conditions, which could, in turn, have long-term health consequences at adult age [6,7,8,9], particularly cardio vascular disease [9]. This is what Barker named “the fetal origins hypothesis” in 1995 [10]. More precisely, Barker explained that small length and weight at birth and disproportion in head size could constitute makers of lack of nutrients or oxygen at particular stages of gestation, thus increasing the risk of coronary heart disease in adulthood, as demonstrated by Osmond et al. in 1993 [11]. 

In this context, a variable named Favorable Fetal Growth Condition (FFGC) has been defined in order to capture a variety of (un)favorable conditions related to environmental, genetic or epigenetic factors, dimensions that are all recognized to play a crucial role in prenatal development. Since the first scientific studies initiated in the 1990s, little attention has been devoted to investigating the existence of such a multi-dimensional variable. Recently, Bollen et al. investigated the possibility that the FFGC could exist and introduced it as a latent variable in a structural equation model in a Filipino islands infant’s cohort [12]. This type of statistical model has the main advantage of allowing the integration of several outcomes instead of considering each in separate models. His findings confirm that the FFGC latent variable mediated the effects of maternal characteristics on several birth outcomes; the model with the latent variable (that measures the indirect effect of maternal characteristics) better fitted the data than a model without it (which assumes a direct effect of maternal characteristics). More recently, a study supported the evidence of a FFGC latent variable on a population of children born in the United States (North Carolina and Pennsylvania) [13]. Replication efforts adopting the same strategy are needed, given that, as discussed by Camerota et al., “robustness is especially important for research on the fetal origins hypothesis, given its possible lifelong implications for human health and development” [13]. 

During the last decade, it was documented that neighborhood characteristics may modify the health impact of risk factors in term of coefficients significance and magnitude [14,15]. To our knowledge, to date, no study has investigated whether the neighborhood socioeconomic level modified the relationship between known risk factors and the FFGC latent variable and the consequences of FFGC on health events.

In this context, one objective of our study was to replicate the approach developed by Bollen et al. (2013) [12] on a population of newborns in Paris, France, and explore whether the model that included the FFGC latent variable better fitted the data. An additional objective was the investigation of the differential effect of the FFGC latent variable according to the parental socioeconomic level measured at the residential census blocks.

## 2. Material and Methods

### 2.1. Study Area 

According to the national census in 2006, Paris, the capital of France, has a population of about 2,250,000 inhabitants and counts about 30,000 newborns per year. The year 2006 was chosen as it was the closest year to the newborn health data. The small-area level used for the analysis is the census block (called IRIS by INSEE, the French National Statistics Institute). These units, the smallest for which socio-demographic data from the census are available, were constructed in order to be as homogeneous as possible in terms of population size and socioeconomical profile. The city of Paris is subdivided into 992 census blocks with a mean population of 2199 inhabitants and a mean area of 0.11 km^2^. 

### 2.2. Individual Data Source

Newborn health data are available from the first birth certificate registered by the Maternal and Child Care department of Paris (named PMI for ‘Protection Maternelle et Infantile’) [14]. This certificate is completed by the health professional before exit of the maternity, within the 8 days following birth, and then sent to the PMI local unit. This database includes all newborns between January 2008 and December 2011. In accordance with the rules regarding protection of personal data, all the residential postal addresses of the mother at the time when the certificate was completed were geocoded at the IRIS level. From this database, we extracted several parental and newborn characteristics defined below.

### 2.3. Parental Characteristics

Several mothers’ characteristics were collected, including age (in years), parity (the number of times that woman had given birth to a fetus with a gestational age >20 weeks), level of education and occupational status. Occupational status was the only available characteristic for the father. Newborn birth at very preterm gestational age (<20 weeks) were excluded from the analysis because the babies only have a very small chance of surviving.

We created three age groups of women according to a threshold defined in the literature [13]: less than 20 years (**younger**) and above 35 years of age (**older**) are known to be two age groups at greater risk of adverse birth outcomes compared to women aged between 20 and 35 years (the referent age group). The initial quantitative parity variable was categorized as a binary variable (first pregnancy: yes or no: **FirstP**). Level of mother education was categorized into four groups: low (**primary school**), intermediate (**secondary**), **middle** (‘baccalauréat’ diploma), and higher education level (chosen as the referent age group given their high number in Paris). The occupational status of mother and father was categorized into two groups only characterizing ‘employed versus unemployed’ (i.e., students or unemployed) parents (**unemployedM** and **unemployedF**, respectively).

### 2.4. Newborn Characteristics 

Three birth outcomes were considered in this study: birth weight (**BW**) in kilograms, length (**BL**) in centimeters and gestational age (**GA**) in weeks. As it is known that birth outcomes are gender differentiated (for instance, mean of birth weight is higher for boys than for girls), infant sex was included as a confounder (**Girl**), with male infants defining the referent group.

### 2.5. Neighborhood Characteristics

To characterize the neighborhood where mothers lived during pregnancy, we chose an estimate of socioeconomic deprivation at the census block scale. The index, based on previous work, was constructed using a principal component analysis (more details are given in Lalloué et al. [16]). A total of 15 socioeconomic and demographic variables collected by the National Institute of Statistics and Economic (INSEE—2012), the most correlated with the first principal component, were selected and linearly combined to estimate the socioeconomic deprivation index for each census block of Paris city. The index was categorized into 10 groups according to the decile of its distribution (named Socio-Economic Status: from **SES1**, the least deprived census blocks to **SES10**, the most deprived census blocks). We choose to categorize into 10 classes of deprivation in order to better capture the large range of socioeconomic inequalities existing in Paris. Thanks to our very large sample size, 10 categories constituted a good compromised to keep enough statistical power and to group homogeneous census blocks in terms of level of deprivation.

A major goal of the current study was to test whether BW, BL, and GA are associated with a common latent variable named the Favorable Fetal Growth Conditions (FFGC), as concluded in Bollen et al. [12], or are three distinct outcomes which have independent relationships with predictors. In this case, we conclude that a model with a latent FFGC variable (indirect-effects model) better fits the data than a direct-effects model. We then stratified the analysis on the level of socioeconomic deprivation with the aim to investigate whether the FFGC always plays the same role and to the same extend in each socioeconomic category.

**Figure 1:**
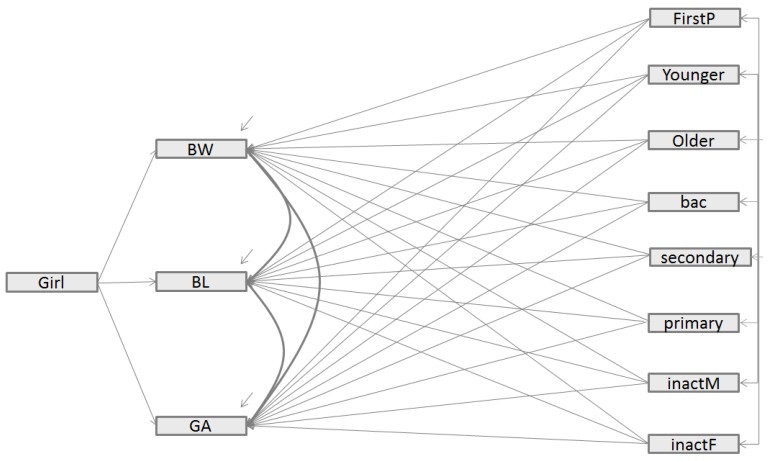
Structural equation model relating parents’ characteristics to those of an infant’s birth (BW, BL, GA) (Model 1). Legend: BW = birth weight; BL = birth length, GA = gestational age; GIRL = newborn is a girl; FirstP = newborn was firstborn; Younger = mother was <20 years old when pregnant; Older = mother was >35 years old when pregnant; primary = women with a low education level; secondary = women with a middle education level; bac = women with a high education level; unemployedM = unemployed mother; unemployedF = unemployed father.

*Direct Effects Model—Unmediated Effects*—The base SEM (Model 1) is depicted in Figure 1. In this model, each predictor has a direct effect on the observed birth outcomes (Birth Weight: **BW**, Birth Length: **BL**, and Gestational Age: **GA**). All the observed predictors’ variables are exogenous (**FirstP, younger, older, Primary, Secondary, bac, unemployedM and unemployed** [see section Parental characteristics for a definition of these variables]). The exogenous variables are allowed to correlate with one another as indicated by the long bar with the short arrows connecting them, except the variable ‘**girl**’; indeed, there is no hypothesis justifying a possible correlation between the sex of the newborn and the occupational status or the level of education, for instance. The arrows drawn between the set of exogenous observed variables and the set of health outcomes indicate the direct influence of one variable on another. Finally, in this model, we allowed errors in **BW**, **BL**, and **GA** to correlate with each other; this means that there is a residual association between the 3 health outcomes not captured by the exogenous variables introduced in the model.

**Figure 2:**
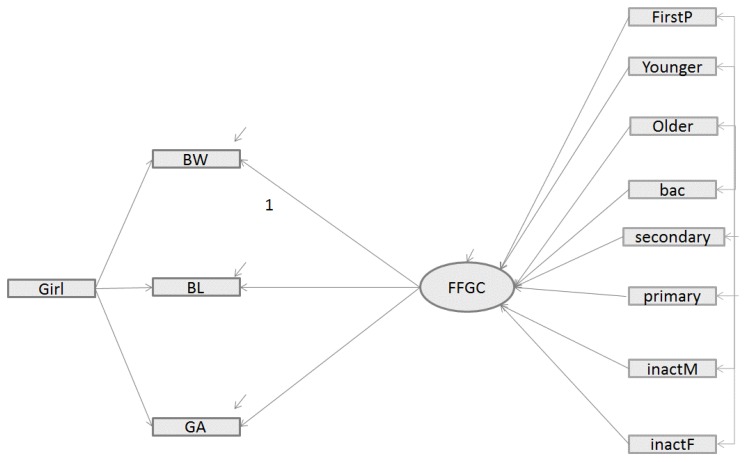
Structural equation model relating parents’ characteristics to those of an infant’s birth (BW, BL, GA) with a mediating latent variable Favorable Fetal Growth Condition (FFGC) (Model 2). Legend: BW= birth weight; BL = birth length, GA = gestational age; GIRL = newborn is a girl; FirstP = newborn was firstborn; Younger = mother was <20 years old when pregnant; Older = mother was >35 years old when pregnant; primary = women with a low education level; secondary = women with a middle education level; bac = women with a high education level; unemployedM = unemployed mother; unemployedF = unemployed father.

*Indirect Effects Model—FFGC as a Mediator*—In the second model (Model 2), a latent variable was added to Model 1 in order to consider possible mediation effects of exogenous variables on BW, BL, and GA, as proposed by Bollen [17]. In Figure 2, the latent variable FFGC is represented with an oval sign and the observed variables with a rectangle. As in previous studies, to assign a scale to the latent variable, we fixed the path between the latent variable FFGC and one outcome, the birth weight to 1 (the standard deviation of the BW variable being the highest). In this model, we hypothesized that the latent variable FFGC constituted an unobserved measure of a blend of favorable or unfavorable conditions for fetal growth, which simultaneously affected BW, BL and GA. The exogenous variables may affect the FFGC variable differently, which could increase or decrease the BW, BL and GA. Only the girl variable did not have a direct effect on FFGC because it constituted an intrinsic characteristic of the baby while other variables characterized the environment for fetuses’ growth. Considering the direct relation of the girl variable on the 3 outcomes allowed us to take into account the greater vulnerability of the male fetus. In contrast with Model 1, this model does not allow error correlations between the outcomes (no residual relationship among them), considering that possible associations between them are already captured by their common dependence on the FFGC latent variable. However, like the first model, all the exogenous observed variables were allowed to correlate with one another, except the variable ‘**girl’**.

**Figure 3:**
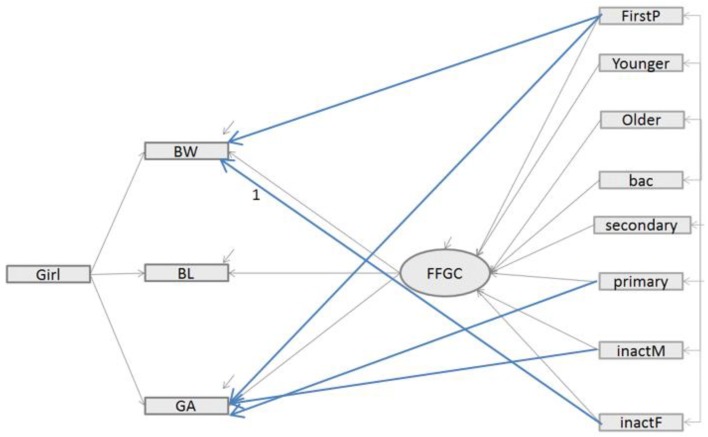
Structural equation model relating parents’ characteristics to those of an infant’s birth (BW, BL, GA) with a mediating latent variable FFGC and additional paths (Model 3). Legend: BW= birth weight; BL = birth length, GA = gestational age; GIRL = newborn is a girl; FirstP = newborn was firstborn; Younger = mother was <20 years old when pregnant; Older = mother was >35 years old when pregnant; primary =women with a low education level; secondary = women with a middle education level; bac = women with a high education level; unemployedM = unemployed mother; unemployedF = unemployed father.

*Modified FFGC Latent Variable Model—Model 3***.** We explored empirical modifications to Model 2, like Camerota and Bollen [13], by adding some omitted paths detected by statistical tests provided by the statistical software (SAS, Using PRoc Calis function). Based on these statistics, we only considered modifications which were plausible and theoretically justifiable. Thus, the plausible paths were the direct path: from FirstP to GA; from FirstP to BW; from unemployedF to BW, from unemployedM to GA and from primary to GA; these additional paths were represented in the following Figure 3 with blue arrows. 

### 2.6. Statistical Analysis

*Imputation of missing values*—The descriptive analysis revealed high rates of missing values for occupational status of the mother and of the father as well as the level of education of the mother (varying between 30% and 40%). As the missing values are not randomly distributed over the study area, we imputed the data based on the census data collected in city of Paris by the National Institute of Statistics and Economic (INSEE—2012). In each census block, we randomly attributed an education level and an occupational status to mothers and fathers with missing values in order to obtain comparable individual and neighborhood distributions in terms of education level and occupational status.

*Statistical procedure*—First, we considered all the newborns in the statistical analysis. Then, we explored potential changes in the model estimates when taking into account the neighborhood deprivation where the mother lived. Thus, we stratified the analysis on ten classes of the neighborhood socioeconomic deprivation index to analyze

*(i)*  whether Model 2 and/or Model 3 fit better than Model 1 in each sub-group, separately, and
*(ii)*  whether the signs, the magnitude and the p-value of the regression coefficients related to FFGC varied between each sub-group (from the most deprived census blocks to the most advantaged).

*Statistical indicators*—We used several statistical indicators to assess overall model fit: the SRMR (Standardized Root Mean Square Residual), the RMSEA (Root Mean Square Error of Approximation), the CFI (Comparative Fit Index) of Bentler, as recommended by O’Rourke N and Hatcher L [18]. The closer [1-RMSEA] and CFI are to 1, the better the model fit. However, CFI close to 0.94 was suggested to define a good fit model [18]. The closer the SRMR is to 0 (or lower than 0.055), the better the model. An additional statistical indicator was used to compare SEM models: the Bayesian information criterion (BIC); a difference of 10 or more in the BICs between two models suggests evidence in favor of the model with the lowest BIC [19]. 

All the models were estimated in SAS using full-information robust maximum likelihood (MLR) as our estimator.

## 3. Results

### 3.1. Population Description 

Descriptive statistics of the population are presented in Table 1. A total of 115,112 births were recorded during the study period 2008–2011. After exclusion of all births with unknown birth weight and gestational age, as well as those with birth weights lower than 500 g, we counted 110,746 singleton births (about 3.8% of the total births were excluded). Then, we also excluded newborns without a maternal address (about 4.9%). Finally, our study considered a total of 105,346 singleton births (91.52%). The mean birth weight of newborns is 3314 g (σ = 499 g) and varied between 540 and 5800 g. The gestational age, on average, was equal to 39 weeks (range = (23–45) weeks). The birth length of newborns varied between 25 and 60 centimeters, with a mean equal to 49.7 centimeters. About 4.6% of births occurred before 37 weeks of pregnancy which corresponds to 4871 preterm births. Concerning parental characteristics, Table 1 shows a low rate of younger compared to older mothers (0.60% vs. 29.49% respectively). Approximately half the mothers were unemployed (45.16%) and half of them had a high level of education (47.1%).

### 3.2. Main Findings of the Structural Equation Models

Our first model’s comparison addressed whether a model with a mediating FFGC latent variable better fit the data than a model with mothers’ and fathers’ characteristics directly influencing birth outcomes (Model 2 versus Model 1). In the second step, we analyzed the results obtained with the indirect model with the FFGC latent variable, including the additional direct path between parental characteristic and BL, BW and GA, compared to those from the model with mothers’ characteristics directly influencing birth outcomes (Model 3 versus Model 1). 

Figure 1, Figure 2 and Figure 3 recapitulate the structure of Models 1, 2 and 3, respectively, including the different links estimated. Like in the Bollen et al. study [12], we reported, in Table 2, global fit indices for the three different SEM models, including the Chi square test statistic from robust maximum likelihood estimator with its corresponding degrees of freedom (df) and p-value, as well as the fit statistics indicators, namely CFI, SRMR, [1-RMSEA] and BIC. 

Overall, Models 1, 2 and 3 all explain a significant part of the birth outcomes variabilities (chi-squares tests are statistically significant *p*-values < 0.001). This was an expected finding, as the sample size was very large (N = 105,346), leading to a high statistical power. 

The analysis of the overall fit statistics for the three models highlighted several interesting findings.

- In Model 2, both CFI and [1-RMSEA] values were close to but below the ideal fit of 1, indicating that Model 2 provided a good fit for the data). However, Model 2 had a higher BIC value than the direct model (Model 1) (981.3 vs. 818.8). According to literature recommendations [19], Model 2 did not provide a better fit to the data because the BIC value of Model 2 was higher that the BIC value of the direct model, even if CFI and [1-RMSEA] were close 1).
- Inversely, in Model 3, the addition of direct paths between exogenous variables and birth outcomes improved the model fit. The fit statistics indicate an excellent fit for the data: as with model 2, the CFI and [1-RMSEA] were close to 1. However, a more substantial gap is visible for BIC which, in Model 3, is lower than for Model 1 (780.7 vs. 818.8) and the difference is greater than 10. Model 3, which includes the FFGC latent variable as a mediator variable between the parents characteristics and the birth outcomes (BW, BL, and GA) and includes additional direct paths, appears to be the best model.

Concerning the interpretation of Model 3, we examined both the sign and the significance of the regression coefficients (Table 3). In this Model 3, all the added paths from fisrtP, girls, unemployedF to BW (β = −0.132, −0.060, −0.002), girl to BL (β = −0.163) and girl, firstP, unemployedM and primary to GA (0.0086, 0.058, −0.01843, −0.01602) were significant at 5%. 

The mother characteristics (FirstP, Older and Younger) had statistically significant negative effects on FFGC. The level of education (bac, secondary, primary) also had a significant negative effect on the latent variable compared to the highest level of education, as well as the occupational status of the father (unemployedF), while, inversely, the occupational status of the mother (unemployedM) was positively associated with FFGC (it is borderline significant: *p*-value = 0.04).

### 3.3. Sensitivity Analysis 

Model 3 always presented the best fit statistics compared to those obtained with Model 1, whatever the neighborhood socioeconomic group. However, the regression coefficients reveal differences in terms of signs, magnitude and p-value between sub-group of socioeconomic deprivation. As Table 4 shows, the variables of parity (FirstP), maternal age (Older, younger) and education level of the mother were negatively related to FFGC for newborns living in the two most deprived census blocks (SES = 10 and SES = 9) as well as the two privileged (SES = 1 and SES = 2). In addition, whereas maternal occupational status was never significantly associated with FFGC, the father’s occupational status (unemployedF) negatively predict FFGC, but only among newborns living in the most deprived socio-economic neighborhood (SES = 10 and SES = 9). 

However, there were two exceptions (see Supplementary Table S1):

- For newborns living in the census blocks categorized in SES8, the measure of parity (FirstP) was not significant; in addition, young mothers and those with a primary level of education were negatively related to FFGC.
- For newborns living in the census blocks categorized in SES7, only the measures of parity (FirstP), maternal age (Older) negatively predicted FFGC (see Supplementary Table S1).

## 4. Discussion

Fetal environment, which is known to be related with pregnancy outcomes [20,21], is a complex notion which cannot be directly observed through a singular and simple way. While it is well documented that several factors play a crucial role in fetal development [22], we could only use parental characteristics in this analysis. Introducing them in an SEM model, we tested the hypothesis that an underlying latent variable, namely the FFGC variable, an unobserved variable, could partially express the effects of this blend of parental characteristics on BW, BL and GA. Parental characteristics, in particular, level of education and occupational status, have been reported to be associated with fetal growth [7]. Indeed, Silva et al. explained that maternal level of educational reflectes material resources (because it partially determines the occupational status and income level) and non-economic social dimensions, including the level of knowledge about health [7], which could impact the birth weight, the head circumference and other birth measures. The mechanisms through which parental traits may be indirectly related to adverse birth outcomes remain unclear. However, we may formulate a hypothesis. These parental characteristics may reflect (1) poor living conditions [23], which could relate to airborne exposure of the fetus, in association with poor housing [24], living near high traffic areas or workplace air pollution, (2) unhealthy food [25] (due to a low level of education and/or to limited resources [7,26]), which could damage or slow fetal growth by lack of essential nutriments [27] and (3) insufficient pregnancy screening [23], which could reduce the chance of early detect infections and other adverse situations. All these factors are separately known to reduce gestational age, birth weight and birth length, representing different dimensions of fetal growth, which might be captured in the favorable/unfavorable fetal growth conditions latent variable. Hence, as recommended by Camerota and Bollen [13], it is important to replicate this indirect model proposed by Bollen et al. [12] in different populations and settings to accumulate evidence for the existence of an FFGC latent variable. According to the fetal origins hypothesis, impairment of birth conditions may have lifelong implications for human health and development [28]. 

The main objective of our study was to apply this novel approach to another population, here newborns in the city of Paris (2008–2011). One original aspect of our study was to investigate whether results found on the overall population concerning the latent variable could vary according to the level of socioeconomic deprivation measured at the residential census blocks of the mothers. Comparing fit statistics from the model where parents’ characteristics directly affected BW, BL, and GA (Model 1) to one in which these effects were mediated by FFGC (Modified FFGC Latent, Model 3), we confirm that the latter provided a better fit for the data. 

One limitation, highlighted by Bollen et al. [12] in their study, was related to the collected data: birth weight and length, and gestational age were self-reported by the mothers and this could introduce bias in the measures of association. In 2016, Camerota and Bollen [13] wanted to overpass this limitation and replicated their study with independent measures of the birth outcomes: one self-reported by the mother and the other reported by health professionals, as in our study. They confirmed that the indirect model (including the FFGC latent variable) better fit the data, as in our finding. Thus, the results appear robust to how information on birth outcomes were collected.

Most interestingly, we stratified our analysis according to the neighborhood socioeconomic index estimated at the census block scale to test: *i)* first, whether the FFGC Model was a better fit in each sub-group, and *ii)* secondly, whether the patterns of signs and significance of regression coefficient to predict FFGC were different between each sub-group. Our study found that a model with a latent FFGC variable fit the data better than a model without it, whatever the class of neighborhood socioeconomic deprivation. Hence, our study suggests that the existence of the latent variable characterizing favorable fetal growth conditions was not undermined by the level of neighborhood socioeconomic deprivation. However, our findings reveal that the strength, the direction and the level of significance of the association between the exogenous variables and the FFGC were different according to the level of neighborhood deprivation. Therefore, our study suggests that mechanisms and/or the strength of the mediating effect of the FFGC on adverse birth outcomes may be influenced by the level of socioeconomic deprivation; further investigations are needed to gather additional scientific evidence.

The strengths and limitations of this study should be addressed here. The main strength of our study is the databases we used, with a high rate of completeness of the birth certificates (93% on average) and the large population size, resulting in a high statistical power when stratifying the statistical analysis by neighborhood deprivation category. However, one limitation related to the stratified analysis is the high number of missing values that we imputed using information available at the census block scale. Doing so, we increased the level of correlation between the distribution of individual characteristics and that of the neighborhood. Hence, we decreased between-subject variability within census blocks, especially among the most deprived census blocks in which the rate of the missing value was twice higher compared to the most privileged ones. However, our results consistently reveal significant relations with the FFGC variable; this may suggest that imputation values did not have a significant influence on this main result but rather much more on the measure of the associations and their level of significance between exogenous variables and the FFGC variable. Additionally, our study presents the advantage of consider three birth outcomes together in the same analysis while the majority of the epidemiological studies investigated each one separately when data of several outcomes were available. A methodological approach taking into account that correlation between the birth outcomes is more realistic, knowing, for instance, that small gestational age reduces the birth weight and height. While the development of the structural equation model has existed for a long time [29,30,31], to date, few studies have dealt with birth outcomes [12,13]. We encourage new research conducted in the perinatal field to adopt this approach.

The main limitation of our study is the absence of certain mothers’ characteristics, such as maternal height and weight (or the body mass index), smoking habits or alcohol consumption during pregnancy; these factors are all known risk factors of adverse birth outcomes. However, we took into account individual and neighborhood socio-economic information, which are documented to be related to unhealthy behaviors; thus, we believe that we partially captured these missing risk factors thanks to these socioeconomic data, and thus minimized the resulting bias. Additional limitation of this study concerns the difficulty to extrapolate our findings to other French areas. Indeed, the Ile de France region is one of the French regions where the proportion of population with a high level of education is the highest (https://www.insee.fr/fr/statistiques/1288219). This may have influenced the association with the latent variable and needs to be investigated in other areas with various socioeconomic profiles. 

In addition, in our study, only one variable, occupational status, was available to characterize the socioeconomic position of the father. In future research, it could be very interesting, of course, to consider the level of education of the father and of the mother in the same analysis. 

## 5. Conclusions

In conclusion, our study supports the existence of a latent variable representing a FFGC-mediating variable between parents’ characteristics and newborn weight, length and gestational age. Future research should aim to confirm the existence of the FFGC latent variable, taking into account neighborhood characteristics using Multi-Level Structural Equations, which combine the analytic approaches of multilevel analysis and structural equation modeling in a two-stage process.

## Figures and Tables

**Table 1:** Descriptive statistics for newborns’ and parents’ characteristics.

	N	Point Estimate	Standard Deviation	Minimum	Maximum
*Newborns’ characteristics*					
Birth weight (grs)	105,346	3314	499	540	5800
Birth length (cms)	102,589	49.7	2.3	25	60
gestational age (weeks)	105,346	39.1	1.6	23	45
gender-girl (%)	105,346	49.3			
*Parents’ characteristics*					
Parity (%)	104,461	39.2			
Unemployed mother (%)	105,346	45.2			
Unemployed father (%)	105,346	37.1			
*Age*	105,346				
younger (<20 years) (%)	632	0.6%			
Middle (20–35 years) (%)	73,637	69.9%			
Older (≥35 years) (%)	31,077	29.5%			
*Level of education*	105,346				
Higher (%)	49,584	47.1%			
bac (%)	6005	5.7%			
Secondary (%)	3867	3.7%			
Primary (%)	45,890	43.6%			

**Table 2:** Global fit measures for structural equation models.

Model	Chi^2^	df	*p*-Value	SRMR	RMSEA	[1-RMSEA]	BIC	CFI
**Model 1**	11.6423	8	<0.0001	0.0012	0.0021	0.9979	818.8	1
**Model 2**	335.5241	22	<0.0001	0.0058	0.0118	0.9882	981.2	0.9988
**Model 3**	88.8684	18	<0.0001	0.0022	0.0062	0.9938	780.7	0.9997

Legend: Model 1: Direct model; Model 2: Indirect Model (FFGC); Model 3: Indirect model + additional direct paths. SRMR: standardized root mean squared residual; RMSEA: Root Mean Square Error of Approximation; BIC: Bayesian Information Criteria; CFI: Confirmatory Fit Index.

**Table 3:** Full-information robust maximum likelihood (MLR) estimates of the direct effects of parents’ characteristics on Favorable Fetal Growth Conditions (FFGC) from Model 3 (as described in Figure 3).

Parents’ Characteristics	β	SE	*p*-Value
FirstP	−0.03771	0.00360	<0.0001
unemployedF	−0.02396	0.00682	0.0004
unemployedM	0.00959	0.00473	0.0425
*Age*			
Younger	−0.01913	0.00335	<0.0001
Middle	ref	---	---
Older	−0.01871	0.00338	<0.0001
*Level of education*			
Higher	ref	---	---
bac	−0.02233	0.00344	<0.0001
Secondary	−0.02971	0.00344	<0.0001
Primary	−0.05511	0.00427	<0.0001

Legends: FirstP = newborn was firstborn; Younger= mother was <20 years old when pregnant; Older = mother was >35 years old when pregnant; primary = women with a low education level; secondary = women with a middle education level; bac = women with a high education level; unemployedM = unemployed mother; unemployedF = unemployed father; SE: Standard Error; β: coefficient of regression.

**Table 4:** MLR estimates of the direct effects of parents’ characteristics on Favorable Fetal Growth Conditions (FFGC) from Model 3 stratified by neighborhood deprivation index (as described in the Figure 3).

	The Least Deprived Census Blocks						The Most Deprived Census Blocks					
Parents’ Charactéristics	SES1 (10,567)			SES2 (10,449)			SES9 (10,523)			SES10 (10,513)		
	β	SE	*p*-Value	β	SE	*p*-Value	β	SE	*p*-Value	β	SE	*p*-Value
Firstp	−0.05445	0.01134	<0.0001	−0.05202	0.01144	<0.0001	−0.04178	0.01129	0.0002	−0.03433	0.01120	0.0022
Unemployedf	0.00630	0.02126	0.7670	−0.01342	0.02176	0.5373	−0.05573	0.02030	0.0061	−0.03872	0.01843	0.0357
Unemployedm	0.02707	0.01392	0.0519	0.02548	0.01418	0.0724	0.00415	0.01430	0.7716	−0.01126	0.01363	0.4087
*Age of the Mother*												
Younger	0.0008263	0.01059	0.9378	−0.00335	0.01066	0.7534	−0.02141	0.01057	0.0429	−0.01962	0.01061	0.0644
Middle	ref	--	--	ref	--	--	ref	--	--	ref	--	--
Older	−0.03073	0.01075	0.0042	−0.02165	0.01084	0.0458	−0.02846	0.01064	0.0075	−0.01248	0.01061	0.2396
*Level of Mother Education*												
Superior	ref	--	--	ref	--	--	ref	--	--	ref	--	--
bac	−0.03120	0.01063	0.0033	−0.01657	0.01075	0.1230	−0.03449	0.01136	0.0024	−0.03278	0.01207	0.0066
Secondary	−0.02233	0.01065	0.0359	−0.01907	0.01077	0.0766	−0.03335	0.01130	0.0032	−0.04767	0.01196	<0.0001
Primary	−0.05553	0.01261	<0.0001	−0.07422	0.01286	<0.0001	−0.05797	0.01384	<0.0001	−0.04571	0.01468	0.0019

Legends: FirstP = newborn was firstborn; Younger= mother was <20 years old when pregnant; Older = mother was >35 years old when pregnant; primary = women with a low education level; secondary = women with a middle education level; bac = women with a high education level; unemployedM = unemployed mother; unemployedF = unemployed father; SE: Standard Error; β: coefficient of regression. SES: socioeconomic Status; in parenthesis: the total number of newborns; Ref: referent group.

## Supplementary Materials

The following are available online at https://www.mdpi.com/1660-4601/16/21/4069/s1, Table S1, MLR estimates of the direct effects of parents’ characteristics on Favorable Fetal Growth Conditions (FFGC) from Model 3 stratified by neighborhood deprivation index (as described in the Figure 3).
