# Supplementary material for: Adverse Birth Outcomes as Indicators of Poor Fetal Growth Conditions in a French Newborn Population—A Stratified Analysis by Neighborhood Deprivation Level
Source: Int J Environ Res Public Health. 2019 Oct 23;16(21):4069. doi: 10.3390/ijerph16214069 (PMC6861961; doi:10.3390/ijerph16214069)
Supplement: Supplementary file 1 [file ijerph-16-04069-s001.pdf]

Table S1: **MLR Estimates of Direct Effects of Parents' Characteristics on Favorable Fetal Growth Conditions (FFGC) from Model 3 stratified by neighborhood deprivation index (as described in the Figure 3)**

	**The least deprived census blocks**									**The most deprived census blocks**								
	**SES3 (10,524)**			**SES4 (10,584)**			**SES5 (10,520)**			**SES6 (10,575)**			**SES7 (10,466)**			**SES8 (10,625)**		
**Parents' Charactéristics**	**B**	**SE**	**p-value**	**B**	**SE**	**p-value**	**B**	**SE**	**p-value**	**B**	**SE**	**p-value**	**B**	**SE**	**p-value**	**B**	**SE**	**p-value**
firstP	-0.03812	0.01131	0.0008	-0.04121	0.01149	0.0003	-0.05020	0.01148	<.0001	-0.04237	0.01142	0.0002	-0.03486	0.01154	0.0025	-0.09782	0.10021	0.3290
unemployedF	-0.01523	0.02187	0.4861	-0.01326	0.02248	0.5553	-0.0006356	0.02200	0.9770	-0.01660	0.02171	0.4447	-0.04089	0.02261	0.0705	-0.08794	0.17440	0.6141
unemployedM	0.01648	0.01463	0.2601	0.01729	0.01483	0.2436	0.01423	0.01527	0.3513	0.01477	0.01522	0.3319	0.01128	0.01561	0.4700	-0.07623	0.12619	0.5458
Age of the mother																		
younger	-0.02728	0.01058	0.0099	-0.01441	0.01054	0.1715	-0.02169	0.01060	0.0408	-0.01727	0.01057	0.1022	-0.01839	0.01062	0.0832	-1.74793	0.56672	0.0020
Middle	ref	- -	--	ref	--	--	ref	--	--	ref	--	--	*ref*	--	--	ref	--	--
Older	-0.01216	0.01074	0.2576	-0.03348	0.01066	0.0017	-0.00593	0.01071	0.5797	-0.02675	0.01071	0.0125	*-0.02078*	*0.01074*	*0.0531*	-0.06515	0.10122	0.5198
Level of mother education																		
Superior	ref	- -	--	ref	--	--	ref	--	--	ref	--	--	*ref*	--	--	ref	--	--
bac	-0.00634	0.01073	0.5544	-0.01748	0.01073	0.1033	-0.03216	0.01082	0.0030	-0.02250	0.01092	0.0393	0.00174	0.01104	0.8745	-0.14363	0.19450	0.4602
secondary	-0.02171	0.01073	0.0431	-0.03101	0.01069	0.0037	-0.02505	0.01089	0.0214	-0.04705	0.01086	<.0001	-0.00414	0.01104	0.7077	-0.30455	0.23254	0.1903
primary	-0.04429	0.01283	0.0006	-0.05282	0.01304	<.0001	-0.05269	0.01354	<.0001	-0.05384	0.01344	<.0001	-0.01208	0.01376	0.3799	-0.30010	0.11727	0.0105

Legends: FirstP = newborn was firstborn; Younger= mother was <20 years old when pregnant; Older = mother was >35 years old when pregnant; primary=Women with low education level; secondary= Women with middle education level; bac= Women with high education level; unemployedM = unemployed mother; unemployedF = unemployed father; SE: Standard Error; : coefficient of regression. SES: socioeconomic Status; Ref: referent group;
